# Risk Biomarkers for Biochemical Recurrence after Radical Prostatectomy for Prostate Cancer Using Clinical and MRI-Derived Semantic Features

**DOI:** 10.3390/cancers15215296

**Published:** 2023-11-05

**Authors:** Adalgisa Guerra, Filipe Caseiro Alves, Kris Maes, Rui Maio, Geert Villeirs, Helena Mouriño

**Affiliations:** 1 Department of Radiology, Hospital da Luz Lisbon, 1500-650 Lisboa, Portugal; rui.maio@hospitaldaluz.pt; 2 Faculty of Medicine, Clinical Research CIBIT/ICNAS, University of Coimbra, 3004-504 Coimbra, Portugal; caseiroalves@gmail.com; 3 Department of Urology, Hospital da Luz Lisbon, 1500-650 Lisboa, Portugal; kmaes@hospitaldaluz.pt; 4 Nova Medical School, Nova University of Lisbon, 1169-056 Lisbon, Portugal; 5 Department of Medical Imaging, Ghent University Hospital, 9000 Ghent, Belgium; geert.villeirs@uzgent.be; 6 CEAUL, Centro de Estatística e Aplicações, Faculdade de Ciências, Universidade de Lisboa, 1749-016 Lisboa, Portugal; mhnunes@fc.ul.pt

**Keywords:** extracapsular extension, prostate cancer, magnetic resonance imaging, radical prostatectomy, staging, biochemical recurrence, biochemical recurrence-free survival

## Abstract

**Simple Summary:**

Multiparametric magnetic resonance imaging (mpMRI) is now standard practice for suspected prostate cancer (PCa) patients, significantly enhancing risk assessment and PCa detection. Integrating MRI into clinical staging allows for more precise, personalized treatment planning in cases of extraprostatic cancer extension. Adverse MRI findings, such as a macroscopic extracapsular extension on MRI (mECE+), capsular disruption, extended tumor capsular contact length (TCCL), Grade Group (GG) ≥ 4, positive surgical margins (PSM), and pECE+ on pathology, were associated with higher biochemical recurrence (BCR) risk. Particularly in low/intermediate-risk patients (pECE− and GG < 4), adverse MRI characteristics correlated with elevated BCR risk. This feature highlights the importance of incorporating predictive MRI features pre-surgery to aid clinical decisions and enhance outcomes in prostate cancer. Adverse MRI features assist in identifying low/intermediate-risk patients needing closer monitoring.

**Abstract:**

Objectives: This study aimed to assess the impact of the covariates derived from a predictive model for detecting extracapsular extension on pathology (pECE+) on biochemical recurrence-free survival (BCRFS) within 4 years after robotic-assisted radical prostatectomy (RARP). Methods: Retrospective data analysis was conducted from a single center between 2015 and 2022. Variables under consideration included prostate-specific antigen (PSA) levels, patient age, prostate volume, MRI semantic features, and Grade Group (GG). We also assessed the influence of pECE+ and positive surgical margins on BCRFS. To attain these goals, we used the Kaplan–Meier survival function and the multivariable Cox regression model. Additionally, we analyzed the MRI features on BCR (biochemical recurrence) in low/intermediate risk patients. Results: A total of 177 participants with a follow-up exceeding 6 months post-RARP were included. The 1-year, 2-year, and 4-year risks of BCR after radical prostatectomy were 5%, 13%, and 21%, respectively. The non-parametric approach for the survival analysis showed that adverse MRI features such as macroscopic ECE on MRI (mECE+), capsular disruption, high tumor capsular contact length (TCCL), GG ≥ 4, positive surgical margins (PSM), and pECE+ on pathology were risk factors for BCR. In low/intermediate-risk patients (pECE− and GG < 4), the presence of adverse MRI features has been shown to increase the risk of BCR. Conclusions: The study highlights the importance of incorporating predictive MRI features for detecting extracapsular extension pre-surgery in influencing early outcomes and clinical decision making; mECE+, TCCL, capsular disruption, and GG ≥ 4 based on pre-surgical biopsy were independent prognostic factors for early BCR. The presence of adverse features on MRI can assist in identifying low/intermediate-risk patients who will benefit from closer monitoring.

## 1. Introduction

Prostate cancer (PCa) represents the second leading cause of death and the most frequent cancer in men worldwide, with over 268,490 new estimated cases diagnosed in 2021 [1]. Between 27% and 53% of all patients undergoing curative radical prostatectomy (RP) or prostate cancer (PCa) radiation therapy (RT) develop a biochemical recurrence (BCR) [2]. The biochemical recurrence, after radical prostatectomy, is defined as PSA > 0.2 ng/mL with a second confirmatory level of prostate-specific antigen of >0.2 ng/mL [3]. BCR can be a surrogate marker of prostate cancer recurrence. However, it is important to note that a rising PSA level does not always mean that cancer has already metastasized and that the natural history of PSA-only recurrence can be prolonged [2,4]. However, a systematic review and meta-analysis that investigated the impact of BCR on outcome endpoints concluded that patients with BCR are at an increased risk of developing distant metastases and cancer-specific mortality [5]. The European Association Guidelines, recommend that patients with pathological ISUP (International Society Urological Pathology) grade 4–5, that is, Grade Group (GG) 4–5, combined with locally advanced disease in the specimen (pT3) and with or without surgical margins, are at high risk for BCR [6] and should be offered adjuvant intervention after prostatectomy. The low/intermediate risk patients’ ISUP 1–3 (GG < 4) and pT2 may not require immediate intervention [7].

Adding mp-MR information may assist clinicians to better stratify patients and accurately predict the outcome of patients with tumors that have spread outside the prostate gland. By incorporating MRI into clinical staging algorithms, clinicians can create more accurate and personalized treatment plans for patients with extraprostatic cancer spread [8,9,10,11,12].

Based on the covariates derived from our previous model [13], we aim to assess the influence of MRI, clinical, and histological features on biochemical recurrence-free survival (BCFS), following prostatectomy in patients with PCa. Additionally, we seek to identify adverse MRI features in patients with a low to intermediate risk of biochemical recurrence.

## 2. Materials and Methods

This prospective single-center study included 228 participants from a previous cohort used to perform and validate a predictive model to detect pECE+ in patients operated by RARP at Hospital da Luz, Lisbon [13]. All patients had a diagnosis of PCa, underwent an MRI exam with a standard protocol [13], and underwent surgery between 2015 and 2020. Each participant was subsequently followed from the date of prostatectomy until May 2022 in order to record the exact date of biochemical recurrence. Fifty-one patients were excluded because they were lost for follow-up (Figure S1).

The outcome of the study, biochemical recurrence-free survival (BCRFS), was defined as the time-lapse between curative prostatectomy and the earliest date of BCR, which was defined as a prostate-specific antigen level of 0.2 ng/mL after an interval of undetectable prostate-specific antigen.

**Figure 1:**
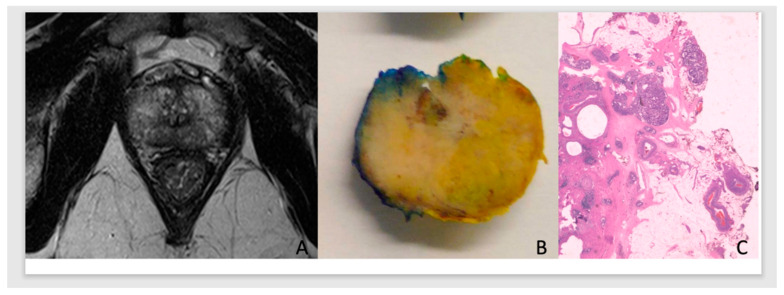
Illustration of the MRI, anatomical, and histology of PCa. ADC prostate G7(3+4)/Grade Group 2, in the apex with low signal on T2WI, high TCCL, and budging on MRI (**A**) on the right apex in the anatomic specimen (**B**), with pECE+ on histology (**C**).

Features:

We used all the covariates from the pECE+ predictive model described in our previous paper [13]. Therefore, the covariates analyzed in this study were as follows:

- Semantic MRI interpretative features set (black striation in periprostatic fat, obliteration of the rectoprostatic angle, measurable ECE on MRI (mECE+), smooth capsular bulging, capsular disruption, indistinct margin, and irregular contour) used for predicting pECE+ on MRI.
- The index lesion length (ILL) corresponds to the major length of the index lesion and the tumor capsular contact length (TCCL), which is the contact length of the index lesion with the prostate capsule. Both were measured in millimeters on axial T2 images, and we used a curvilinear ruler to draw the TCCL.
- PI-RADS V2 for the characterization of the index lesion [14].
- Gleason score (GS)/Grade Group (GG) on the prostate specimen. The GG was divided into low/intermediate risk ISUP 1-3 (GS ≤ 4+3) or GG < 4; and high risk, ISUP 4-5 (GS ≥ 4+4) or GG ≥ 4, for BCR, according to the literature [7,15].
- The clinical and laboratory data evaluated included the age of the patients, PSA levels at surgery, PSA density (PSA/prostate volume), and MRI and surgery dates. Patients’ data were anonymized, collected in an Excel database, and organized according to the surgery dates. Categorization of the PSA: PSA < 6 ng/mL, 6 ng/mL ≤ PSA < 10 ng/mL, and PSA ≥ 10 ng/mL.
- In this predictive analysis, we added PCa pathological staging and surgical margins results of the prostate specimen [13]. Tumors were classified as pECE negative (pECE−) if no tumoral cells were detected on extracapsular tissue, and pECE positive (pECE+) if the presence of a tumoral extension beyond the periphery of the prostate gland was detected (Figure 1). Positive surgical margins (PSM) refer to the presence of tumor cells beyond the inked surgical margins of the resected tumor.

### Statistical Analysis

We conducted exploratory data analysis, including descriptive statistics and hypothesis testing, to compare patients with and without biochemical recurrence using risk features identified by Guerra et al. [13]. Statistical tests included two-sample z-tests, Fisher’s exact tests, and the Fisher–Freeman–Halton test. The Kaplan–Meier method and log-rank tests were used to compare survival curves. Univariable and multivariable Cox proportional hazard models were applied, highlighting hazard ratios and 95% confidence intervals. We estimated survival curves for low/intermediate-risk and high-risk ISUP patients and examined the effect of mECE+ and pECE+ on biochemical recurrence risk. The analyses were conducted using the R package. Unless otherwise stated, the level of significance considered in the statistical analyses is 5%.

## 3. Results

### 3.1. Exploratory Analysis

Table 1 displays the characteristics of the patients according to the presence of biochemical recurrence (BCR+ or BCR−): 23% were BCR+ and 77% BCR (BCR−) after prostatectomy. In the exploratory analysis, all variables introduced in the previous predictive model to detect pECE+ were significantly different (here we considered *p*-values < 0.10) between BCR+ patients and BCR− patients, except the age of the participants. Patients with BCR+ had more extensive lesions, larger TCCL, higher PSA levels, smaller prostate size, and a higher PSAD ratio. Most patients with BCR+ had a PI-RADS score of 5 (75%). The majority of patients with BCR+ (82.5%) had GG < 4 (ISUP 1-3); it is worth stressing that there were only 17 individuals in the whole sample (9.6% of the total) with GG ≥ 4 (ISUP > 3). The early semantic features for the prediction of pECE+ as smooth capsular bulging, indistinct margins, irregular contour, and capsular disruption were present more often in BCR+ patients than in BCR− patients (roughly, the percentage of BCR+ patients with each of these features was double than that of BCR− patients). On the other hand, 89.8%, 71.5%, and 76.6% of the patients with BCR− did not manifest mECE+, PSMm, and pECE+, respectively.

Of low/intermediate risk patients (112), with GG < 4 and pECE−, 15 patients (13%) were BCR+ and 97 patients (87%) were BCR−. The mean of TCCL and tumor size were higher in the BCR+ group (TCCL: 12.5 mm versus 8.4 mm; index lesion size: 14.8 mm versus 12.1 mm), and there were statistical differences between the two groups, as there were with the individual semantic MRI features smooth capsular bulging, capsular disruption, irregular contour and PI-RADS score (Table 2).

### 3.2. Survival Analysis

**Figure 2:**
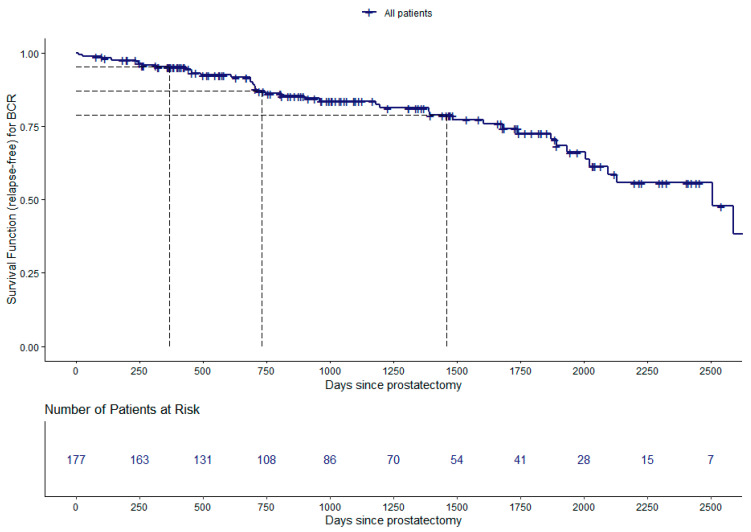
Estimation of the survival curve for the biochemical recurrence-free survival (BCRFS) study: Kaplan–Meier survival function (relapse-free); number of patients at risk for every 250 days; the dashed lines represent the estimates for the survival curve at 365, 730, and 1460 days.

**Figure 3:**
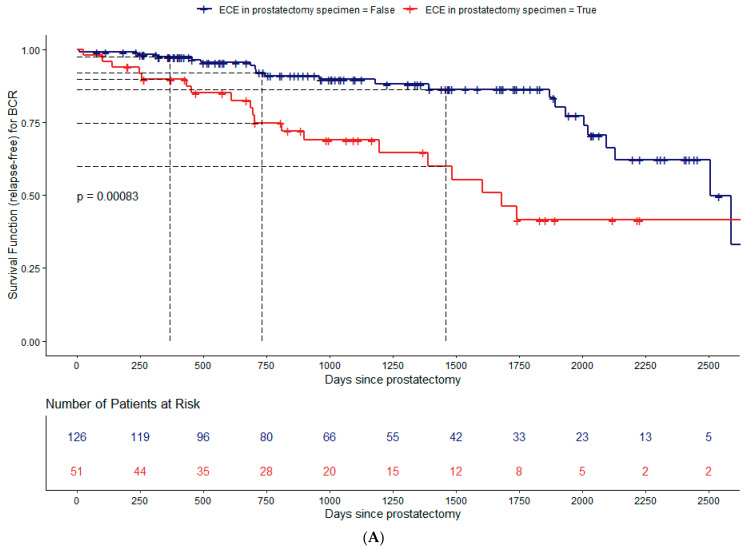

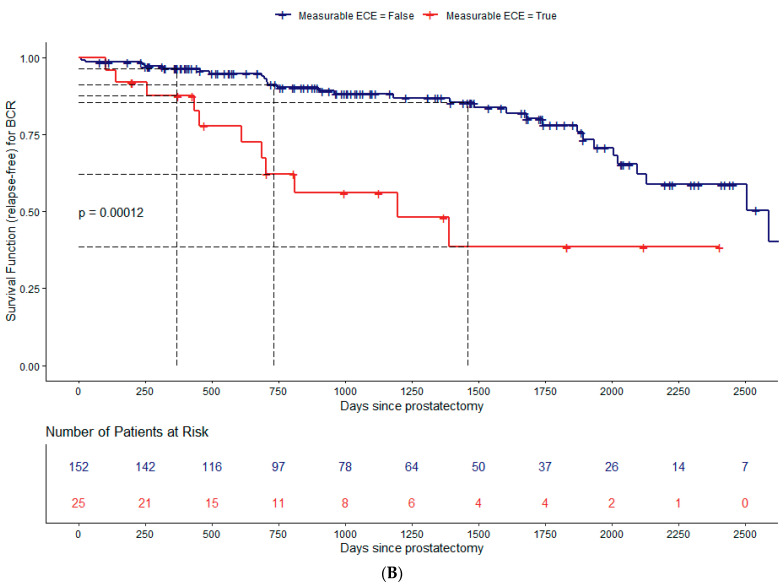

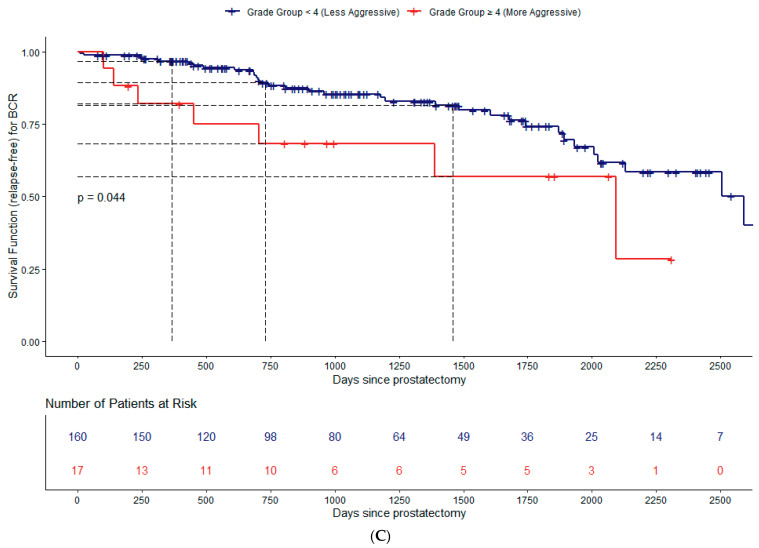

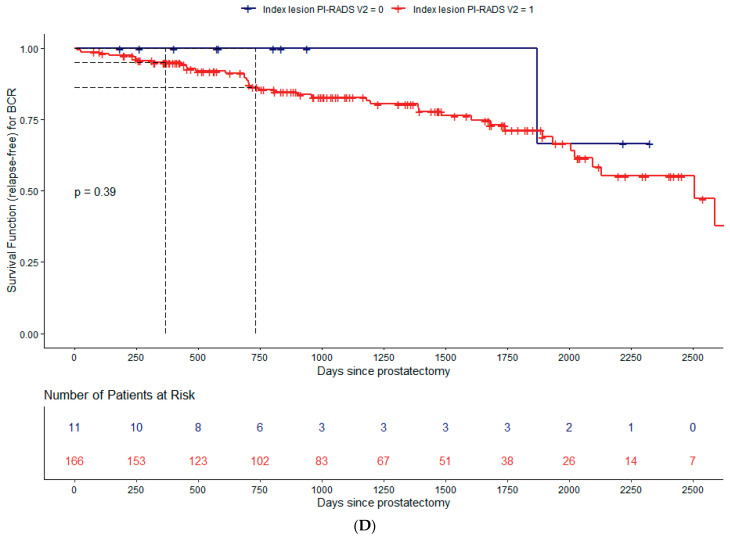

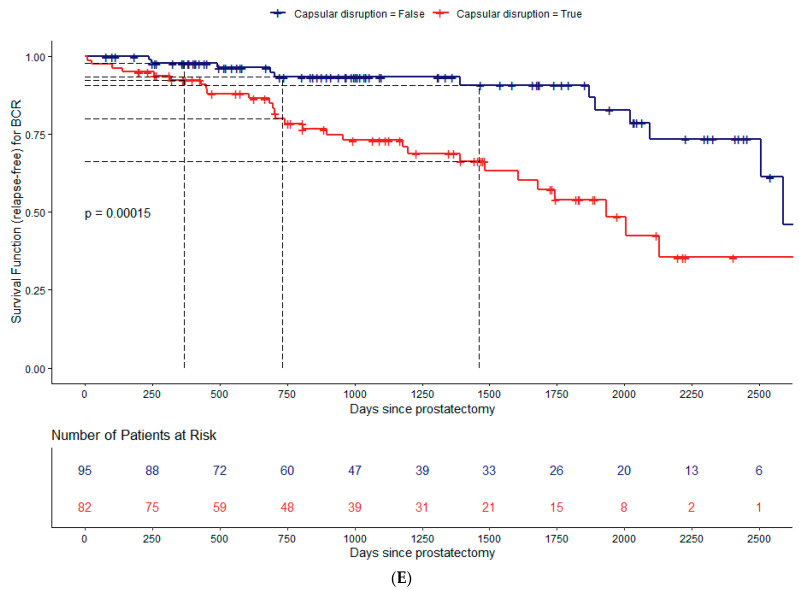

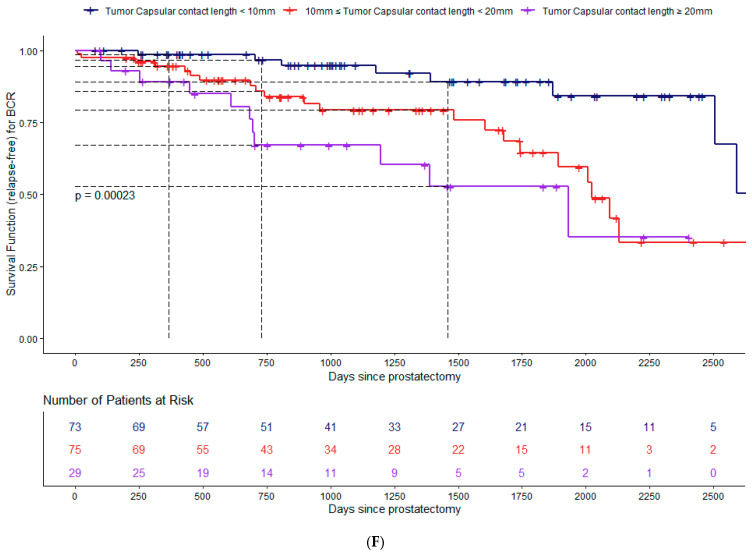

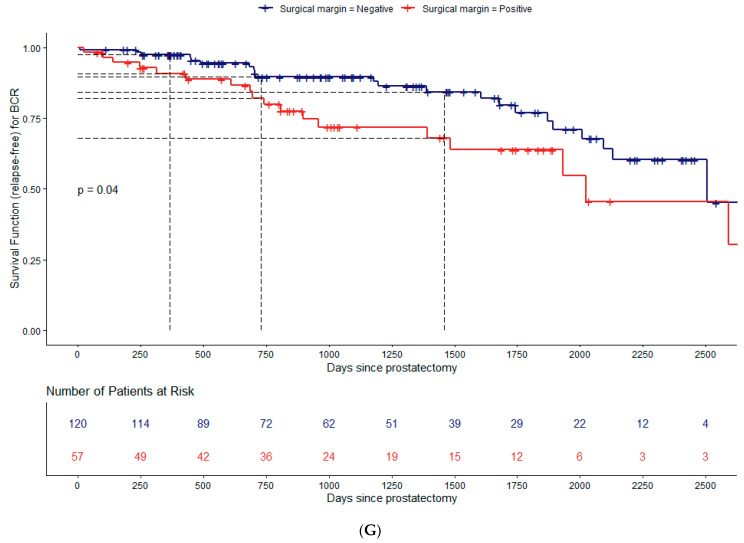

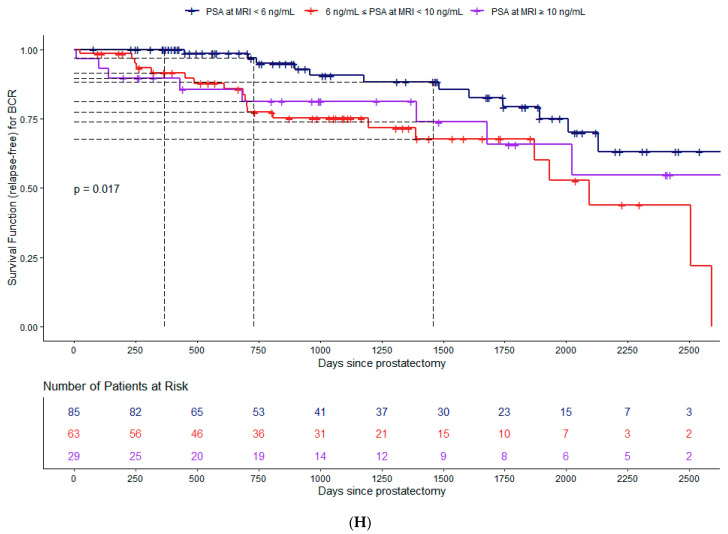
Estimation of the survival curves for the biochemical recurrence-free survival (BCRFS) study. Kaplan–Meier survival function (relapse-free) stratified using pECE based on pathologic specimen staging (**A**), measurable ECE (**B**), Grade Group: GG < 4 (less aggressive) *versus* GG ≥ 4 (more aggressive) (**C**), index lesion PI-RADS.V2: PI-RADS.V2 = 3 versus PI-RADS V2 ≥ 4 (**D**), capsular disruption (**E**), TCCL (**F**), surgical margins (**G**), and PSA (**H**); number of patients at risk for every 250 days; *p*-value from the two-tailed log-rank test to compare the survival curves. The dashed lines represent the estimates for the survival curves at 365, 730, and 1460 days. These estimates were not made for the index lesion PIRADS.V2 due to the reduced number of observations. All categorical variables are divided into two strata: present (red line) *versus* not present (blue line). For the continuous variables, they were divided as follows. Categorization of the TCCL: 0, if TCCL < 10 mm (blue line); 1, if 10 mm ≤ TCCL < 20 mm (red line); 2, if TCCL ≥ 20 mm (purple line). Categorization of the PSA: 0, if PSA < 6 ng/mL (blue line); 1, if 6 ng/mL ≤ PSA < 10 ng/mL (red line); 2, if PSA ≥ 10 ng/mL (purple line).

We analyzed the time between curative intent prostatectomy and biochemical recurrence (BCRFS). The main results are depicted in Figure 2 and Figure 3 and Table 3. The Kaplan–Meier estimate of the survival function for the global BCRFS is illustrated in Figure 2. The estimates of BCRFS probability after curative prostatectomy were 95%, with 95% CI: (92, 99), 87%, with 95% CI: (82, 93), and 79%, with 95% CI: (72, 87), at 1, 2, and 4 years, respectively (Figure 2 and Table 3).

We also estimated the survival curves for each categorical covariate under study. The goal was to evaluate the extent to which the survival curves differ across the categories of the covariates.

The results of the Kaplan–Meier estimate for the survival functions (BCRFS) stratified using pECE, measurable ECE on MRI, GG low/intermediate (GG < 4) *versus* high (GG ≥ 4) risk, index lesion PIRADS v2, capsular disruption, TCCL, surgical margins, and PSA levels categorized are illustrated in Figure 3A–H, respectively, and Table 3. The log-rank test was used to compare the survival curves of the different strata for each covariate cited above.

At the 5% significance level, there are statistical differences between the two survival curves for BCRFS when stratified using all variables (*p*-values < 0.05). The only exception is for index lesion PIRADS v2 (Figure 3D).

The greater the TCCL, the higher the cumulative probability of biochemical recurrence. It is important to notice that the estimated cumulative probability of a patient’s recurrence with TCCL ≥ 20 mm at one year of follow-up is the same (11%) as a patient’s recurrence with TCCL < 10 mm at four years of follow-up.

Patients with PSM have a higher risk for BCR than those with NSM, which increases over time (Figure 3G, Table 3). The estimated BCRFS probability is 91% for patients with PSM in the first-year post-surgery and 68% at four years of follow-up.

In our previous study (13), the GS ≥ 7(4+3), which included grade groups 3, 4, and 5, was identified as a relevant biomarker for pECE+. However, in our current preliminary analysis, only the GS ≥ 8 (Grade Group 4–5), emerged as a relevant risk factor to BCR (*p*-value = 0.044 for the log-rank test).

**Figure 4:**
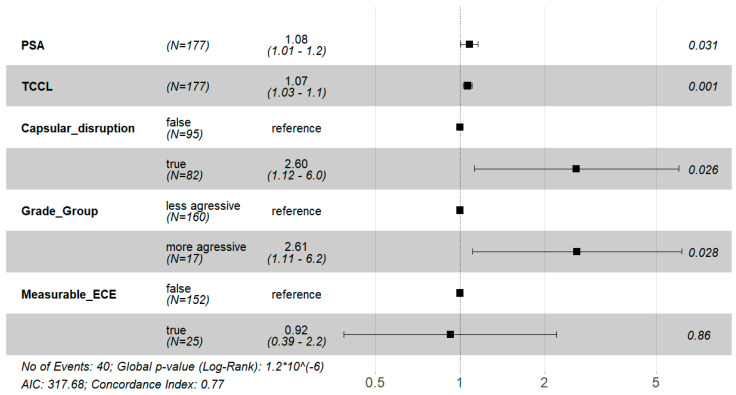
Forest Plot from the multivariable Cox proportional hazards regression model with the covariates PSA, TCCL, CD, GG: GG < 4 (less aggressive) *versus* GG ≥ 4 (more aggressive), mECE+: Hazard Ratio (HR) (black squares) and respective 95% Confidence Interval, CI, (solid horizontal lines) for each covariate; *p*-values (last column). The vertical dashed line HR = 1 is represented in the figure. If the horizontal line of the CI crosses the line HR = 1, the respective covariate is not statistically significant. Number of events, global *p*-value to evaluate the overall significance of the model based on the log-rank test, AIC, and the concordance index are also shown in the figure.

We fitted the multivariable Cox regression model to evaluate the effect of the semantic and clinical covariates on the time until biochemical recurrence. The main results are shown in Table S1 and Figure 4.

The multivariable Cox regression model showed that PSA, TCCL, capsular disruption, and Grade Group were significant risk factors for biochemical recurrence (BCR) (*p* < 0.05) because the respective hazard ratios are statistically greater than one. More precisely, a one-unit increase in PSA leads to an 8% increase in the expected hazard of BCR, keeping the remaining covariates constant (HR = 1.08, 95% CI: [1.01, 1.20]); a one-unit increase in TCCL leads to a 7% increase in the expected hazard of BCR, keeping the remaining covariates constant (HR = 1.07, 95% CI:[1.03, 1.10]); patients presenting capsular disruption have 2.6 times higher expected hazard of having BCR compared to patients without capsular disruption, holding the other variables constant (HR = 2.6, 95% CI:[1.12, 6.00]); patients with more aggressive GG have 2.61 times higher expected hazard of having BCR compared to patients without capsular disruption, holding the remaining variables constant (HR = 2.61, 95% CI:[1.11, 6.20]).

Regarding the Cox regression model, the assumption of proportional hazards is satisfied (*p*-value = 0.309). In terms of the goodness-of-fit measures (Figure 4), the *p*-value for the global statistical significance of the model, based on the Log-Rank statistic, is approximately zero, which means the variables included in the Cox regression model are relevant to describe the time until BCR. The concordance index (c-index) [16], which measures the predictive information derived from the fitted model, is 0.77 (95% CI:[0.69, 0.85]), which shows that the fitted model has good discriminatory power in distinguishing long-term BCRs from short-term ones.

## 4. Discussion

Multiparametric MRI has been acknowledged as a dependable and valuable imaging technique for the detection and precise localization of PCa, and it is also effective in safely ruling out clinically significant PCa in biopsy-naïve individuals and those who have previously had negative biopsies [17].

In this study, researchers aimed to investigate the relationship between the relevant variables from a previously developed predictive model for detecting extracapsular extension (pECE+) (13) on MRI and early-term oncologic outcomes, specifically biochemical recurrence (BCR) up to four years after prostatectomy. The study also aimed to analyze the MRI features that affect the probability of disease recurrence in low/intermediate-risk patients.

The study demonstrated that the prognostic features for detecting pECE+ on MRI, such as the presence of mECE+ (visible on MRI), capsular disruption, and high tumor contact length (TCCL), also impacted BCR+ as demonstrated in the survival analysis. Patients without these signs on MRI (mECE-, no capsular disruption, and TCCL < 10 mm) had a lower risk factor for BCR+. Other early MRI semantic features are individually important but were not discriminatory in the statistical analysis.

On the other hand, patients with macroscopic extracapsular extension (mECE+) have a worse prognosis than those with pathologically confirmed extracapsular extension (pECE+). This means that when ECE is not visible on MRI, it is a favorable prognostic factor, even though it cannot guarantee the absence of microscopic pECE+. Moreover, recent literature has shown that local MRI staging is an independent risk factor for long-term oncologic outcomes, including BCR+, the development of metastatic disease, and prostate cancer-related mortality [18]. The observation that MRI findings predictive of pECE+ indicate risk regardless of histological results might contribute to the ongoing refinement of clinical prostate cancer algorithms. By redefining risk groups using MRI findings instead of digital rectal examination (DRE) findings, better BCR-free survival can be achieved due to improved discrimination of non-organ-confined disease. This strategy could have important implications for treatment planning, neoadjuvant therapies, and monitoring, although more information is needed regarding disease recurrence, PSA-specific mortality, and overall survival (OS).

In this study, only the GG ≥ 4 were considered histological risk factors for BCR. It aligns with European guidelines [4], which did not consider GG ≤ 3 as a high-risk factor for BCR.

Although PSA was not identified as a predictive feature for pECE+ in the previous model [13], its value should be considered as a biomarker of poor prognosis for BCR before surgery. Elevated PSA levels are associated with more aggressive disease and indicate an increased risk for biochemical recurrence.

This study further underscores the importance of classic prognostic biomarkers such as pECE+, PSM, PSA, and high-risk GG in established prognostication tools following prostatectomy, as supported by previous research [5,19,20,21,22]. However, this model enables us to observe that even patients without these risk characteristics for BCR+, commonly referred to as low/intermediate-risk patients (pECE−, GG < 4, can potentially benefit from pre-surgery MRI to evaluate adverse staging MRI features (high TCCL and tumor size, smooth capsular bulging capsular disruption, capsular disruption, and PI-RADS score). These MRI features confer a certain level of risk and should be considered when managing these patients. If the study were intended for external validation, it might be appropriate to consider shorter established follow-up intervals after RP for patients with adverse staging MRI features mentioned previously.

The extrapolation of the timing of biochemical recurrence (BCR) and death in prostate cancer (PCa) is not well established. Previous studies have shown that longer times to BCR after radical prostatectomy (RP) are associated with a higher likelihood of localized disease and decreased PCa mortality [23]. However, more recent studies have failed to find a consistent association between time to BCR and death from PCa [24]. Various variables, such as GS, pathological stage, surgical margin status, and lymph node involvement, are related to BCR and should be considered to predict local or distant recurrence. Short PSA doubling time (mainly PSA-DT < 6 months), GG ≥ 4, seminal vesicle invasion (SVI) (pT3b), and lymph node positivity appear to be the main factors associated with metastatic disease and PCa mortality. Therefore, stratifying men with PCa into risk groups is crucial for defining prognosis and treatment decisions [24].

It is important to acknowledge several limitations of our study. First, we only analyzed the early outcome of BCR, and further analysis is needed to assess the model’s influence on PCa disease progression and mortality. In this study, PSMA PET was not considered, even though this technology has been utilized for detecting metastatic disease [25], particularly in high-risk patients. It could be intriguing to assess the impact of PSMA PET on biochemical recurrence (BCR) in both high-risk and low-risk patients.

Our cohort was limited to a single institution and a single therapeutic approach (robot-assisted radical prostatectomy—RARP), which might restrict the generalization of our findings to other institutions and other management options such as radiation therapy (RT), focal therapy, or active surveillance. Future research might focus on a multi-center study. Furthermore, a more extensive timeline should be considered to accurately determine an interval estimate for the five-year survival probability (besides the one-, two-, and four-year survival probabilities reported here) due to the increased number of patients under consideration.

We did not evaluate the influence of seminal vesicle invasion (SVI) separately from extracapsular extension (pECE+) in our analysis. Additionally, we did not consider lymph node metastasis and the impact of adjuvant RT on post-surgical outcomes. The amount of positive surgical margins (PSM) was also not considered, although it varied between 1 cm and 1 mm, with a mean of less than 5 mm on pathology examination.

Further research is needed to understand better the prognostic significance of our predictive model in long-term disease progression-free survival and the influence of other neoadjuvant therapeutics used in cases of positive surgical margins immediately after prostatectomy. In our study, the MRI features likely correlate with biochemical or genetic changes, heightening the risk of biochemical recurrence (BCR). Gaining a deeper comprehension of the connection between these MRI features and the probability of BCR, particularly when considering genetic data, could hold significant promise for future research endeavors.

Finally, a recent prospective study [26] has demonstrated that the new artificial intelligence tools offer a promising avenue for a personalized treatment approach for prostate cancer patients. These techniques will give valuable insights into paving the way for future research in this area.

## 5. Conclusions

This study suggests that in addition to the important role of pathologic tumor stage as a prognostic factor, the predictive MRI features for detecting extracapsular extension (ECE) before surgery also significantly impact early outcomes and should be taken into consideration in clinical decision making. The presence of macroscopic ECE (visible on MRI), tumor contact length (TCCL), capsular disruption, and GG ≥ 4 can be regarded as independent prognostic factors for early biochemical recurrence (BCR). It is particularly important to determine the adverse staging MRI features in low/intermediate-risk patients (pECE−, GG < 4) to identify individuals who require closer monitoring. By incorporating these factors into the clinical assessment, healthcare professionals can identify patients who may benefit from more intensive follow-up and potentially adjuvant intervention strategies.

## Figures and Tables

**Table 1:** Characteristics of the patients based on Biochemical Recurrence, BCR (sample size = 177).

Variables	BCR+ (Nº of Patients = 40)	BCR− (Nº of Patients = 137)	*p*-Value
Continuous variables			
Age at MRI (years)	61.5 ± 5.6 (51.7; 73.0)	61.3 ± 6.8 (41.2; 75.2)	0.845
Prostate volume (g)	36.6 ± 12.1 (20; 86)	44.9 ± 21.9 (19; 150)	0.002
PSA (ng/mL)	8.0 ± 4.0 (2.6; 20.0)	6.6 ± 3.4 (2.2; 21.2)	0.038
PSAD * (ng/mL/g)	0.23 ± 0.10 (0.06; 0.50)	0.17 ± 0.12 (0.04; 0.96)	0.003
Index lesion size (mm)	17.4 ± 6.6 (7.0; 39.0)	13.3 ± 5.2 (5.0; 30.0)	0.000
Tumor capsular contact length (mm)	17.3 ± 10.6 (0.0; 57.0)	10.6 ± 7.6 (0.0; 35.0)	0.000
Categorical variables			
Index lesion PI-RADS V2			
3	1 (2.50)	10 (7.30)	0.000
4	9 (22.50)	83 (60.58)	
5	30 (75.00)	44 (32.12)	
Smooth capsular bulging			
No	8 (20.00)	72 (52.55)	0.001
Yes	32 (80.00)	65 (47.45)	
Capsular disruption			
No	12 (30.00)	83 (60.58)	0.001
Yes	28 (70.00)	54 (39.42)	
Indistinct margin			
No	11 (27.50)	79 (57.66)	0.001
Yes	29 (72.50)	58 (42.34)	
Irregular contour			
No	13 (32.50)	91 (66.42)	0.000
Yes	27 (67.50)	46 (33.58)	
Black striation in periprostatic fat			
No	26 (65.00)	113 (82.48)	0.027
Yes	14 (35.00)	24 (17.52)	
Measurable ECE			
No	29 (72.50)	123 (89.78)	0.010
Yes	11 (27.50)	14 (10.22)	
ECE in prostatectomy specimen **			
No	21 (52.50)	105 (76.64)	0.005
Yes	19 (47.50)	32 (23.36)	
Retoprostatic angle obliteration			
No	34 (85.00)	132 (96.35)	0.018
Yes	6 (15.00)	5 (3.65)	
Surgical margins			
Negative	22 (55.00)	98 (71.53)	0.076
Positive	18 (45.00)	39 (28.47)	
Grade Group (GG)			
GG < 4	33 (82.50)	127 (92.70)	0.068
GG ≥ 4	7 (17.50)	10 (7.30)	

Each continuous variable is represented as the average ± standard deviation (minimum; maximum). Each categorical variable is described by the number of patients in each level (percentage). The *p*-values were obtained by the following tests: two-tailed Fisher’s exact tests or its extension (Fisher–Freeman–Halton test) for categorical variables; two-sample z-test (two-tailed tests) for continuous variables. * PSAD: PSA density, i.e., PSAD = PSA/Prostate Volume; ** gold standard.

**Table 2:** Characteristics of low/intermediate-risk patients based on Biochemical Recurrence, BCR (sample size = 112).

Variables	BCR+ (Nº of Patients = 15)	BCR− (Nº of Patients = 97)	*p*-Value
Continuous variables			
Prostate volume (g)	38.2 ± 14.2 (24; 86)	45.8 ± 22.0 (19; 122)	0.120
PSA (ng/dL)	6.7 ± 3.4 (2.6; 14.0)	6.4 ± 3.2 (2.2; 20.7)	0.704
Index lesion size (mm)	14.8 ± 4.4 (7.0; 22.0)	12.1 ± 4.5 (5.0; 30.0)	0.019
Tumor capsular contact length (mm)	12.5 ± 6.7 (0.0; 23.0)	8.4 ± 6.1 (0.0; 24.0)	0.021
Categorical variables			
Index lesion PI-RADS V2			
3	1 (6.70)	8 (8.25)	0.016
4	5 (33.33)	65 (67.01)	
5	9 (60.00)	24 (24.74)	
Smooth capsular bulging			
No	4 (26.67)	59 (60.82)	0.023
Yes	11 (73.33)	38 (39.18)	
Capsular disruption			
No	7 (46.67)	72 (74.23)	0.037
Yes	8 (53.33)	25 (25.77)	
Indistinct margin			
No	7 (46.67)	67 (69.07)	0.140
Yes	8 (53.33)	30 (30.93)	
Irregular contour			
No	8 (53.33)	77 (79.38)	0.047
Yes	7 (46.67)	20 (20.62)	
Black striation in periprostatic fat			
No	13 (86.67)	88 (90.72)	0.640
Yes	2 (13.33)	9 (9.28)	
Measurable ECE			
No	15 (100.00)	95 (97.94)	NA
Yes	0 (0.00)	2 (2.06)	
Retoprostatic angle obliteration			
No	15 (100.00)	97 (100.00)	NA
Yes	0 (0.00)	0 (0.00)	

Low/intermediate-risk Patient: Grade Group < 4 and pECE negative. NA: Not Available. Due to the lack of data, it was not possible to perform the corresponding statistical test. Each continuous variable is represented as the average ± standard deviation (minimum; maximum). Each categorical variable is described by the number of patients in each level (percentage). The *p*-values were obtained by the following tests: two-tailed Fisher’s exact tests or its extension (Fisher–Freeman–Halton test) for categorical variables; Mann–Whitney test (two-tailed tests) for continuous variables.

**Table 3:** Results from the Kaplan–Meier survival curves for each feature under study: Biochemical recurrence-free survival at 1, 2, and 4 years (95% CI); *p*-values from the log-rank tests to compare the survival curves from the groups considered in each feature.

Feature		Biochemical Recurrence-Free Survival			Log-Rank Test *p*-Value
		1-Year (95% CI) *	2-Year (95% CI) *	4-Year (95% CI) *	
pECE−		98 (95, 100)	92 (87, 98)	87 (79, 94)	0.00083
pECE+		90 (82, 99)	75 (63, 89)	60 (45, 80)	
mECE−		97 (94, 100)	91 (87, 96)	86 (79, 93)	0.00012
mECE+		88 (75, 100)	62 (45, 87)	39 (20, 75)	
Grade Group (GG)					
GG < 4		97 (94, 100)	89 (84, 95)	81 (74, 89)	0.04400
GG ≥ 4		82 (65, 100)	68 (49, 96)	57 (35, 93)	
Capsular disruption	Not Present	98 (95, 100)	94 (88, 99)	91 (84, 99)	0.00015
	Present	93 (87, 99)	80 (71, 90)	66 (55, 80)	
Negative Surgical Margin		97 (95, 100)	90 (84, 96)	84 (77, 93)	0.04000
Positive Surgical Margin		91 (83, 99)	82 (72, 94)	68 (55, 84)	
TCCL < 10 mm		99 (96, 100)	97 (92, 100)	89 (80, 99)	0.00023
10 mm ≤ TCCL < 20 mm		95 (89, 100)	86 (77, 95)	79 (69, 91)	
TCCL ≥ 20 mm		89 (78, 100)	67 (51, 89)	53 (34, 82)	
PSA < 6 ng/mL		100 (100, 100)	97 (93, 100)	88 (80, 98)	
6 ng/mL ≤ PSA < 10 ng/mL		92 (85, 99)	77 (67, 90)	68 (55, 84)	0.01700
PSA ≥ 10 ng/mL		90 (79, 100)	81 (68, 98)	74 (57, 96)	
No Strata (all patients)		95 (92, 99)	87 (82, 93)	79 (72, 87)	—

* Values in percentage. TCCL: Tumour Capsular Contact Length. CI: Confidence Interval.

## Data Availability

The data presented in this study are available upon request from the corresponding author.

## Supplementary Materials

The following supporting information can be downloaded at: https://www.mdpi.com/article/10.3390/cancers15215296/s1, Figure S1: Flowchart of the patient selection process; Table S1: Results from fitting a multivariable Cox proportional hazards regression model.
