# Supplementary material for: Risk Biomarkers for Biochemical Recurrence after Radical Prostatectomy for Prostate Cancer Using Clinical and MRI-Derived Semantic Features
Source: Cancers (Basel). 2023 Nov 5;15(21):5296. doi: 10.3390/cancers15215296 (PMC10650512; doi:10.3390/cancers15215296)
Supplement: Supplementary file 1 [file cancers-15-05296-s001.zip › cancers-2671587-supplementary.pdf]

Risk biomarkers for biochemical recurrence after radical prostatectomy for prostate cancer using clinical and MRI-derived semantic features

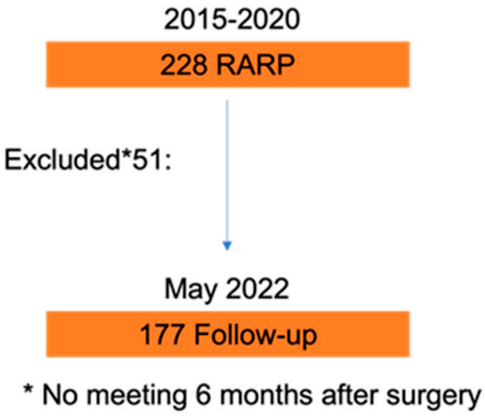

Figure S1. Flowchart of the patient selection process

Table S1. Results from fitting a multivariable Cox proportional hazards regression model.

**Variable**	**Coefficient**	**SE**	**Hazard Ratio (95% CI)**	***p*-value**
Prostate Specific Antigen, PSA	0.078	0.036	1.081 (1.007, 1.161)	0.031
Capsular contact length	0.063	0.019	1.065 (1.026, 1.106)	0.001
Capsular disruption				
Yes	0.955	0.428	2.598 (1.123, 6.011)	0.026
Measurable ECE				
Yes	-0.078	0.445	0.925 (0.387, 2.212)	0.860
Grade Group (GG) high risk				
GG ≥ 4	0.959	0.438	2.609 (1.106, 6.152)	0.028

CI: Confidence Interval
